# Association of specific nutritional intake with periodontitis

**DOI:** 10.1186/s12903-024-04384-6

**Published:** 2024-05-30

**Authors:** Alfonso Varela-López, Beatriz Bullon, Isabel Gallardo, Jose Luis Quiles, Pedro Bullon

**Affiliations:** 1 Department of Physiology, Institute of Nutrition and Food Technology “Jose Mataix”, Biomedical Research Center, University of Granada, Granada, Spain; 2 Department of Stomalogy, Dental School, University of Sevilla, C/Avicena s.n., Sevilla, 41009 Spain

**Keywords:** Diet, Periodontitis, Macronutrients, Fiber, Vitamin, Minerals

## Abstract

**Background:**

The present study aimed to evaluate nutritional intake among a group of male patients in the dental clinic with and without periodontal disease to search for associations between nutritional profile and periodontal health.

**Methods:**

To this purpose, nutritional intake of macronutrients, fiber, vitamins, and minerals were compared evaluating both clinical parameters and periodontal status. Non periodontitis patients were compared with stage III and IV periodontitis and its extension according to the 2017 classification.

**Results:**

After multivariate analysis, statistically significant associations were found between the dietary intake of energy, total fat, cholesterol, calcium, saturated fat, monounsaturated fat and folic acid and iodine and periodontitis status. This study reports an inverse association between cholesterol and iodine and periodontitis and a direct association with saturated fat, monounsaturated fat, and folic acid.

**Conclusions:**

Maintaining an adequate intake of fat, iodine, calcium, and cholesterol and avoiding an excessive intake of energy, saturated fat, monounsaturated fat, and folic acid could be important to controlling periodontitis.

## Background

Various factors have been associated with the etiology of periodontal disease. Undoubtedly, oral microorganisms are indispensable for the pathogenesis of periodontal disease. Therefore, the primary cause is poor oral hygiene, leading to the formation of dental plaque containing microorganisms [1]. In addition to these local elements, several systemic factors appear to influence periodontal health, many of them shared with systemic conditions such as cardiovascular disease [2], and obesity [3]. In this sense, there is a set of well-established risk factors for periodontitis that include alcohol and drug abuse, stress, smoking, genetics, and hormonal alterations [4]. Similarly, the nutritional status of the host has been widely recognized as a possible promoting factor in many inflammatory conditions such as periodontal diseases [5]. In fact, nutritional factors are of vital importance for the equilibrium between oral microorganisms and the host response, which depends on the onset and progression of periodontal disease [6]. The relationship of nutrition and oral health is well known and in recent years a large number of reviews have been published that highlight the link between nutrition and periodontal disease in recent years [5, 7]. However, the results of the epidemiological studies still present differences.

Currently, standardized periodontal therapy is based on manual supragingival and subgingival debridement, with additional supportive antibiotic therapy in strictly defined clinical situations. However, once periodontitis is diagnosed, a common claim is to consider additional benefits of controlling the nutritional status of patients in combination with periodontal therapy [8]. The aim of the study was to evaluate nutritional intake among a group of male patients with and without periodontitis in different states to look for associations between nutritional profile and periodontal health.

## Methods

### Patients

Male subjects over 35 years of age attending the Dental School of the University of Sevilla, who have not experienced a prior cardiac event, and who had no significant coronary disease attending the Dental School of the University of Sevilla (Control) without clinical records of cardiac events, diabetes or periodontal disease diagnosis and treatment were asked to participate in the study. Informed consent was obtained from each subject, the study protocol was in accordance with the ethical guidelines of the Declaration of Helsinki of 1975 and was approved by the Local Research Committee (1588-N-20, 12-21-2016).

### Clinical examination

#### General and lifestyle data

Demographic characteristics (age, height, and weight), medical record (heart rate (HR), diastolic blood pressure (DPB), systolic blood pressure (DPB), waist circumference (WC)), and body mass index (BMI) were recorded. BMI was calculated from standardized measurements of weight and height (weight in kg divided by height in square meters). The Minnesota Leisure Time Physical activity [9] was used to evaluate the level of physical activity. The average weekly exercise hours were obtained asking for the hours spent walking. Smoking status was obtained asking if they are a current smoker (someone who smoke at least one cigarette per day) a former smoker (someone who do not smoke now but smoked in the past) or a never-smoker (someone who has never smoked). Non-smoker were considered any of both, never-smoker and former smoker.

#### Oral examination

The number of remaining teeth was recorded. The following periodontal parameters were recorded: clinical probing depth (PD) as the distance between the gingival margin and the bottom of the pocket, clinical attachment level (CAL) as the distance between the cemento–enamel junction and the bottom of the pocket at six sites per tooth; bleeding on probing (BOP) was also recorded after 10 s at four sites [10]. All periodontal measures were evaluated by the same examiner, whose evaluations of the first ten subjects were calibrated for reproducibility prior to the study; an intra-subject correlation coefficient of 92% was found for PD.

The patients were classified as periodontitis patients and non-periodontitis patients (NoP) and periodontitis patient, respectively, if they were or not within the stage III and IV definition established in the 2017 World Workshop on the Classification of Periodontal and Peri-Implant Diseases and Conditions periodontitis [11]. In turn, periodontitis patients were subdivided into localized periodontitis (LP) generalized periodontitis patients (GP) according to the extent and distribution criteria, LP was defined when < 30% of teeth were involved, whereas GP was defined when ≥ 30% of teeth were involved [11]. Furthermore, mean PD, mean CAL and the proportion of sites with PD ≥ 4 mm and CAL ≥ 5 mm were calculated.

### Assessment of dietary intake

Dietary intake was estimated using a 91-item semiquantitative food frequency questionnaire (FFQ). This FFQ that includes foods from 13 different groups (meats, fish, eggs, legumes, cereals, dairy products, fats, vegetables, fruits, sweets and cakes, drinks, nuts, sauces, and others) was based on previously developed and validated questionnaires for the Spanish population [12]. An additional section provides information on the use of nutritional or dietary supplements. The FFQ items represent mainly a single food, but sometimes comprised of sets of commonly associated foods. Participants were asked to indicate the number of portions of these foods they consumed per day, week, month, or year during the past year. The portion sizes quantified by household measures (i.e., one cup) were explained item by item in the FFQ. Standard serving sizes and food models [13–16] were provided as a reference to aid participants in estimating portion size. A trained person helped the participants complete the FFQ when necessary. All values were then converted to dairy consumption frequencies, and this was multiplied by the size of the portion to calculate the grams of food consumed per day. Nutritional and energy intakes were calculated by multiplying the frequency of consumption of each food item (in grams consumed per day) by the mean content of energy and each nutrient (calculated for the food item concerned) per 100 g of product according to the Food Composition Database by Mataix et al. [17] The daily intake of nutrients for individual FFQ items was then summed to obtain the daily intake of each nutrient or energy. All nutrient and food intakes were then adjusted for energy using the residual method.

### Statistical analysis

All statistical analyses were performed using IBM SPSS 25.0 Statistics software. Data were presented as mean ± standard deviation (SD) or percentage or as median ± interquartile rank (IQR) if the variable does not have a normal distribution. The Kolmogorov-Smirnov test has been used to verify the normal distribution of the quantitative data. ANOVA test for parametric distributions and Mann-Whitney U and Kruskal-Wallis test for non-parametric distribution have been used. The post hoc test utilized for the parametric distribution was Student’s t test with the previous Levine test on the homogeneity of variances, and for the nonparametric distribution, the Mann–Whitney U test was used. Categorical variables were analyzed with a chi-square test to determine the groups that make a difference. Statistical significances with p-values of 0.05 or less were considered. Binary logistic regression analysis was used to analyze the probability of LP and GP according to the intake of caloric intake and the different evaluated nutrient normalized and non-normalized by total energy intake. Similar models adjusted for confounders were also performed. Confounders were selected by performing logistic regression analysis models to calculate the probability of localized and generalized periodontitis using the Backward LR method that considers all target variables, which were all those factors whose values were statistically significant different between groups of patients. Finally, only smoking status was considered for the subsequent adjusted models. Moreover, unadjusted and adjusted ordinal logistic regression models with diagnosis of periodontitis with three ordered categories (non-periodontitis and localized and generalized periodontitis) were also performed.

## Results

### General data

Table 1 shows the mean and SD or median and IQR values of the general data, anthropometrical measures, and physical activity of the sample population. A total of 112 subjects were enrolled in the study, 59 attendants received a diagnosis of NoP, 13 were LP and 40 were GP. GP patients were younger than those with NoP and LP. GP also had lower values of BMI and walking hours values than NoP, while the HR and DBP values were higher. Table 2 shows smoking status with more current smoker in GP than in NoP.

**Table 1:** General data, anthropometrical measures, and physical activity

	NoP (*n* = 59)	LP (*n* = 13)	GP (*n* = 40)
	Mean ± SD Median ± IQR	Mean ± SD Median ± IQR	Mean ± SD Median ± IQR
Age (years)	62.00 ± 15.00^c^	64.54 ± 7.16^c^	58.00 ± 9.00^ab^
Weight (g)	83.69 ± 12.57	77.77 ± 9.29	79.79 ± 12.50
Height (cm)	171.83 ± 7.03	170.23 ± 6.78	172.83 ± 6.30
BMI (g/cm^2^)	27.46 ± 4.59^c^	26.88 ± 3.24	26.74 ± 4.14^a^
WC (cm)	108.05 ± 10.94	104.08 ± 9.05	104.43 ± 11.14
SBP (mmHg)	145.92 ± 23.41	146.3 ± 29.85	149.20 ± 24.63
DBP (mmHg)	82.19 ± 14.28^c^	76.23 ± 10.99	86.45 ± 16.15 ^a^
HR (bpm)	67.00 ± 14.00^c^	68.00 ± 12.00	75.05 ± 11.93^a^
Walking (h)	7.00 ± 6.50^c^	6.08 ± 4.31	4.00 ± 6.25^a^

*Abbreviations* BMI: body mass index, BOP: bleeding on probing, bpm: beat per minute, CAL: clinical attachment level, DBP: diastolic blood pressure, GP: generalized periodontitis, HR: heart rate, IQR: interquartile rank, NoP: non periodontitis, LP: localized periodontitis, PD: probing pocket depth, SBP: systolic blood pressure, SD: standard deviation, WC: waist circumference

^a, b,c,^ indicate statistical differences between groups when they are different

**Table 2:** Absolute (n) and relative (%) frequencies of participant with different smoking status

		NoP (*n* = 59)	LP (*n* = 13)	GP (*n* = 40)
Current smoker		6 (10.2%)^c^	4 (30.8%)	14 (35.0%)^a^
Non-smoker		53 (89.8%)	9 (69.2%)	25 (62.5%)
	Former smoker	29 (49.2%)	8 (61.5%)	23 (51.1%)
	Never-smoker	24 (40.7%)	1 (7.7%)	3 (7.5%)

*Abbreviations* GP: generalized periodontitis, NoP: non periodontitis, LP: localized periodontitis

^a,b,c,^ indicate statistical differences between groups when they are different

### Oral health

The mean values of different clinical periodontal health parameters, including the number of remaining teeth, percentages of teeth with BOP, mean PD, percentages of sites with PD ≥ 4 mm, mean CAL and percentages of sites with CAL ≥ 5 mm are presented in Table 3. BOP was high in GP compared to NoP. The number of remaining teeth was only lower in LP compared to NoP and GP. The values of PD and CAL show the highest values in GP than LP and the lowest in NoP.

**Table 3:** Clinical periodontal health parameters of mild to generalized periodontitis and no periodontitis patients

	NoP (*n* = 59)	LP (*n* = 13)	GP (*n* = 40)
	Mean ± SD Median ± IQR	Mean ± SD Median ± IQR	Mean ± SD Median ± IQR
Teeth	22.03 ± 4.78^b^	17.31 ± 6.07^ac^	20.93 ± 6.56^b^
BOP(%sites)	4.86 ± 6.06^c^	11.66 ± 13.27	19.03 ± 19.41^a^
Mean PD	2.47 ± 0.2^c^	2.58 ± 0.19	3.13 ± 0.37^a^
PD ≥ 4 mm (%sites)	0.04 ± 0.06^bc^	0.10 ± 0.06^ac^	0.38 ± 0.15^ab^
Mean CAL	2.79 ± 0.39^bc^	3.85 ± 1.10^a^	4.65 ± 1.2^a^
CAL ≥ 5 mm (%sites)	0.06 ± 0.07^bc^	0.29 ± 0.24^a^	0.44 ± 0.25^a^

*Abbreviations* BOP: bleeding on probing, CAL: clinical attachment level, GP: generalized periodontitis, IQR: interquartile rank, NoP: non periodontitis, LP: localized periodontitis, PD: probing pocket depth, SD: standard deviation

^a, b,c,^ indicate statistical differences between groups when they are different

### Dietary intake and periodontal health

Table 4 shows the values of oral health parameters according to dietary intake of nutrients only for nutrients that show statistically some significant difference among dietary intake tertiles in the value of some the parameters. No differences were obtained except for two nutrients in one parameter each one. Mean PD value was lower in the lowest tercile of intake of saturated fat normalized by energy intake respect than the second tercile. Regarding the percentage of sites with BOP, the individuals in the lowest tercile of iron intake had a lower value than those in the highest, but this was not observed if the intakes were adjusted by energy consumption.

**Table 4:** Periodontal health parameters values according nutritional intakes (Only values showing statistically significant differences for those between intake are included)

Nutrient	Normalized by energy intake?	Tertile	% BOP Mean ± SD Median ± IQR	Mean PD Mean ± SD Median ± IQR	% sites with PD ≥ 4 Median ± IQR	Mean CAL Mean ± SD Median ± IQR
Saturated fat						
	No	T1	4.17 ± 15.94	2.63 ± 0.44	0.05 ± 0.19	2.85 ± 1.02
		T2	5.87 ± 11.65	2.73 ± 0.32	0.10 ± 0.29	3.20 ± 1.71
		T3	4.17 ± 12.70	2.67 ± 0.41	0.09 ± 0.23	3.21 ± 1.53
	Yes	T1	4.92 ± 13.27	2.71 ± 0.50^b^	0.07 ± 0.18	2.89 ± 1.43
		T2	5.95 ± 16.13	2.77 ± 0.40^a^	0.14 ± 0.34	3.23 ± 1.76
		T3	5.00 ± 10.06	2.65 ± 0.46	0.09 ± 0.25	3.21 ± 1.64
		T1	4.17 ± 15.94	2.63 ± 0.44	0.05 ± 0.19	2.85 ± 1.02
Iron						
	No	T1	3.91 ± 11.14^c^	2.78 ± 0.47	0.14 ± 0.39	3.20 ± 1.51
		T2	5.83 ± 16.72	2.59 ± 0.44	0.07 ± 0.27	3.09 ± 1.68
		T3	5.51 ± 12.59^a^	2.65 ± 0.30	0.09 ± 0.16	3.17 ± 1.56
	Yes	T1	2.56 ± 12.88	2.78 ± 0.43	0.11 ± 0.37	3.68 ± 0.99
		T2	4.49 ± 11.67	2.59 ± 0.36	0.07 ± 0.20	2.93 ± 0.93
		T3	5.76 ± 17.37	2.63 ± 0.45	0.09 ± 0.19	3.11 ± 1.92

*Abbreviations* BOP: bleeding on probing, IQR: interquartile rank, PD: probing pocket depth, CAL: clinical attachment level, T1: first (the lowest) tertile of intake, SD: standard deviation, T2: second tertile of intake, T3: third tertile (the highest) of intake

^a, b,c,^ indicates statistical differences between tertiles when they are different

Table 5 shows the results of the regression analysis for dietary intakes of nutrients showing any statistically significant association. The unadjusted logistic model, increased odds of having GP were associated with the lowest tercile 1 of non-normalized intakes of carbohydrates, fiber, potassium, vitamin A, B1 B2, B6, B9 and C and normalized intakes of fiber, potassium, and vitamin C, compared to the highest consumption tercile. Similar associations were found for the second tercile of non-normalized intakes of vitamin A, C, B1 B2, B6 and potassium and normalized fiber intake. Although the odds of having GP calculated for the lowest tercile 1 of intake of phosphorus, calcium, and energy respect than the highest terciles were not statistically significant, an increased odds was found for the second terciles. Terciles 1 and 2 of fiber, potassium and, vitamin C consumption normalized by energy intake and tercile 1 of energy-intake-normalized consumption of fiber and potassium were associated with localized to generalized periodontitis prevalence with increased odds compared to the highest terciles. In the model adjusted by smoking status, only associations between GP prevalence and non-normalized intake of carbohydrates, vitamin A, B2, B6, C and iron remained statistically significant. Both the lowest and second tercile of phosphorus consumption non-normalized by caloric intake were also associated. In case of potassium and fiber intake, only the association with the lowest tercile 1 remained significant. Among those normalized by energy intake, only the lowest terciles of fiber, vitamin C, and potassium consumption were associated with generalized periodontitis prevalence.

**Table 5:** Associations between dietary intakes and periodontal disease status

Nutrient	Normalized by energy intake?		OR (95%CI) of LP			OR (95%CI) of GP		OR (95%CI) of periodontitis severity	
			T1 vs. T3 (the highest)	T2 vs. T3 (the highest)		T1 vs. T3 (the highest)	T2 vs. T3 (the highest)	T1 vs. T3 (the highest)	T2 vs. T3 (the highest)
Energy		Unadj.	1.05 (0.42, 2.62)	1.81 (0.72, 4.51)		2.03 (0.72, 5.69)	3.96 (1.44, 10.89)*	1.79 (0.23, 13.99)	7.75 (1.00, 59.83)*
		Adj.	1.13 (0.41, 3.11)	2.01 (0.73, 5.52)		2.31 (0.77, 6.95)	4.73 (1.59, 14.03)*	3.30 (0.35, 30.84)	13.49 (1.46, 124.28)*
Carbohydrates	no	Unadj.	1.62 (0.65, 4.03)	1.17 (0.47, 2.91)		3.55 (1.29, 9.77)*	2.28 (0.82, 6.36)	5.90 (0.77, 45.40)	2.34 (0.30, 18.14)
		Adj.	1.18 (0.43, 3.29)	1.15 (0.42, 3.16)		3.01 (1.03, 8.79)*	2.44 (0.82, 7.26)	5.19 (0.57, 47.70)	3.29 (0.36, 30.31)
	yes	Unadj.	1.46 (0.58, 3.67)	2.51 (0.99, 6.37)		1.70 (0.64, 4.55)	2.13 (0.81.5.64)	2.76 (0.35, 21.68)	6.89 (0.89, 53.70)
		Adj.	0.98 (0.35, 2.73)	1.94 (0.69, 5.41)		1.25 (0.44, 3.57)	1.65 (0.59.4.64)	1.38 (0.15, 12.87)	4.33 (0.48, 39.39)
Fiber	no	Unadj.	1.80 (0.72, 4.51)	1.05 (0.42, 2.62)		3.96 (1.44, 10.89)**	2.03 (0.72, 5.70)	7.75 (1.00, 59.83)*	1.79 (0.23, 13.99)
		Adj.	1.08 (0.39, 3.04)	0.96 (0.35, 2.63)		2.93 (1.00, 8.58)	2.03 (0.69, 5.98)	4.51 (0.48, 41.98)	2.01 (0.22, 18.35)
	yes	Unadj.	3.57 (1.38, 9.26)*	1.04 (0.41, 2.66)*		4.23 (1.57, 11.39)**	1.19 (0.42, 3.38)*	22.05 (2.73, 178.21)**	1.20 (0.15, 9.92)
		Adj.	2.35 (0.83, 6.62)	0.86 (0.31, 2.40)		3.02 (1.06, 8.57)*	1.04 (0.35, 3.11)	9.53 (1.04, 87.69)*	0.96 (0.10, 9.15)
Vitamin A	no	Unadj.	1.17 (0.47, 2.91)	1.62 (0.65, 4.03)		3.02 (1.06, 8.63)*	4.20 (1.48, 1.91)**	2.86 (0.37, 22.24)	6.42 (0.83, 49.75)
		Adj.	0.94 (0.34, 2.66)	1.48 (0.54, 4.00)		2.83 (0.92, 8.66)	4.26 (1.42, 12.79)**	3.23 (0.34, 30.80)	7.74 (0.86, 69.55)
	yes	Unadj.	1.30 (0.53, 3.23)	1.05 (0.42, 2.61)		2.20 (0.81, 5.94)	2.46 (0.91, 6.61)	2.74 (0.36, 20.82)	2.32 (0.30, 17.63)
		Adj.	1.06 (0.39, 2.91)	0.91 (0.33, 2.49)		1.96 (0.69, 5.62)	2.43 (0.85, 6.95)	2.50 (0.28, 22.14)	2.49 (0.28, 22.04)
Vitamin C	no	Unadj.	1.45 (0.58, 3.61)	1.30 (0.52, 3.24)		3.76 (1.32, 10.70)*	3.37 (1.18, 9.61)*	4.90 (0.63, 37.94)	3.74 (0.48, 29.03)
		Adj.	1.17 (0.42, 3.25)	1.13 (0.42, 3.05)		3.50 (1.15, 10.64)*	3.28 (1.10, 9.78)*	5.33 (0.56, 50.25)	4.15 (0.46, 37.13)
	yes	Unadj.	2.51 (0.99, 6.37)	1.46 (0.58, 3.67)		3.55 (1.29, 9.77)*	2.28 (0.82, 6.36)	10.74 (1.35, 85.21)*	3.30 (0.41, 26.31)
		Adj.	2.02 (0.73, 5.62)	1.26 (0.46, 3.45)		3.00 (1.03, 8.72)*	2.10 (0.72, 6.15)	9.15 (0.98, 85.12)	3.00 (0.32, 27.72)
Vitamin	no	Unadj.	1.45 (0.58, 3.63)	1.80 (0.72, 4.51)		3.37 (1.18, 9.61)*	3.76 (1.32, 10.70)*	4.43 (0.56, 34.75)	6.64 (0.85, 52.01)
B1		Adj.	1.42 (0.52, 3.88)	1.90 (0.69, 5.24)		3.57 (1.18, 10.81)*	4.16 (1.38, 12.61)*	6.82 (0.73, 63.40)	10.23 (1.10, 95.58)*
	yes	Unadj.	2.26 (0.89, 5.74)	2.26 (0.89, 5.74)		1.87 (0.72, 4.87)	1.33 (0.50, 3.52)	5.90 (0.75, 46.34)	4.16 (0.53, 32.73)
		Adj.	1.46 (0.52, 4.09)	1.89 (0.68, 5.30)		1.25 (0.44, 3.50)	1.05 (0.37, 2.98)	5.51 (0.24, 20.94)	2.57 (0.28, 23.72)
Vitamin	no	Unadj.	1.81 (0.72, 4.55)	2.02 (0.80, 5.08)		3.37 (1.18, 9.61)*	3.76 (1.32, 10.70)*	6.10 (0.77, 48.55)	7.94 (1.00, 63.16)
B2		Adj.	2.05 (0.74, 5.73)	1.89 (0.68, 5.20)		3.97 (1.29, 12.21)*	3.77 (1.24, 11.40)*	11.14 (1.15, 107.65)	8.49 (0.91, 79.35)
	yes	Unadj.	1.80 (0.72, 4.51)	1.05 (0.42, 2.62)		2.05 (0.80, 5.25)	0.80 (0.30, 2.18)	4.63 (0.61, 34.86)	0.91 (0.12, 7.06)
		Adj.	1.99 (0.71, 5.55)	1.79 (0.62, 5.21)		2.20 (0.80, 6.06)	1.18 (0.39, 3.52)	5.51 (0.62, 49.17)	2.66 (0.27, 26.61)
Vitamin	no	Unadj.	2.51 (0.99, 6.37)	1.46 (0.58, 3.67)		6.27 (2.12, 18.58)*	3.25 (1.08, 9.72)*	17.10 (2.11, 138.68)*	3.91 (0.48, 31.55)
B6		Adj.	2.09 (0.75, 5.80)	1.34 (0.49, 3.66)		5.75 (1.84, 17.95)*	3.22 (1.02, 10.11)*	18.11 (1.88, 174.17)*	4.33 (0.46, 40.89)
	yes	Unadj.	2.52 (0.99, 6.37)	1.46 (0.58, 3.67)		2.38 (0.90, 6.27)	1.52 (0.56, 4.08)	7.83 (1.00, 61.09)*	2.47 (0.31, 19.50)
		Adj.	2.31 (0.84, 6.36)	1.49 (0.54, 4.15)		2.16 (0.78, 6.00)	1.52 (0.53, 4.36)	6.94 (0.78, 61.75)	2.63 (0.28, 24.38)
Vitamin	no	Unadj.	1.62 (0.65, 4.04)	1.62 (0.65, 4.04)		3.19 (1.16, 8.78)*	2.56 (0.92, 7.09)	5.32 (0.68, 41.44)	4.15 (0.53, 32.33)
B9		Adj.	1.25 (0.44, 3.60)	1.34 (0.50, 3.62)		1.90 (0.66, 5.42)	1.50 (0.52, 4.29)	5.20 (0.52, 51.46)	3.26 (0.37, 29.01)
	yes	Unadj.	1.57 (0.56, 4.39)	0.82 (0.29, 2.28)		1.65 (0.64, 4.24)	1.04 (0.39, 2.74)	4.31 (0.57, 32.75)	1.67 (0.22, 12.87)
		Adj.	1.80 (0.72, 4.51)	1.05 (0.42, 2.62)		2.13 (0.81, 5.64)	1.70 (0.64, 4.55)	3.86 (0.42, 35.67)	1.27 (0.14, 11.76)
Calcium	no	Unadj.	1.17(0.47,2.91)		1.62(0.65,4.03)	2.56(0.92,7.09)	3.19(1.16,8.78)*	2.62(0.34,20.31)	5.15(0.67,39.63)
		Adj.	1.15(0.41,3.21)		1.70(0.62,4.64)	2.77(0.92,8.27)	3.60(1.22,10.68)*	3.92(0.42,36.79)	6.93(0.77,62.59)
	yes	Unadj.	2.01(0.80,5.05)		1.30(0.52,3.26)	2.33(0.90,6.03)	1.04(0.38,2.81)	6.04(0.79,46.19)	1.53(0.20,11.91)
		Adj.	1.98(0.72,5.48)		1.31(0.48,3.58)	2.29(0.83,6.34)	1.01(0.35,2.88)	5.64(0.63,50.07)	1.48(0.16,13.28)
Phosphorus	no	Unadj.	1.30 (0.52,3.26)		2.01 (0.80,5.05)	2.56 (0.92.7.09)	3.19 (1.16,8.78)*	3.10 (0.40,24.29)	6.93 (0.89,53.99)
		Adj.	1.56 (0.56,4.41)		1.87 (0.69,5.09)	3.21 (1.06,9.72)*	3.13 (1.07,9.13)*	6.85 (0.70,66.90)	7.02 (0.78,63.01)
	yes	Unadj.	2.02 (0.80,5.06)		0.94 (0.37,2.35)	2.59 (1.00,6.72)	0.91 (0.33,2.49)	6.67 (0.88,50.73)	0.85 (0.11,6.68)
		Adj.	2.28 (0.82,6.35)		0.97 (0.35,2.68)	2.86 (1.03,7.97)	0.92 (0.31,2.69)	8.55 (0.96,76.43)	0.96 (0.10,8.84)
Iron	no	Unadj.	1.45 (0.59,3.61)		0.94 (0.38,2.34)	3.55 (1.29,9.77)*	2.28 (0.82,6.36)	5.04 (0.66,38.39)	1.70 (0.22,13.17)
		Adj.	1.33 (0.47,3.74)		0.88 (0.33,2.36)	3.66 (1.23,10.94)*	2.35 (0.80,6.86)	7.35 (0.77,70.46)	2.16 (0.24,19.09)
	yes	Unadj.	1.81 (0.72,4.53)		0.75 (0.30,1.89)	2.29 (0.89,5.85)	0.70 (0.25,1.92)	5.17 (0.69,38.75)	0.50 (0.06,3.96)
		Adj.	1.81 (0.72,4.53)		0.75 (0.30,1.89)	1.90 (0.70,5.13)	0.69 (0.24,2.03)	3.36 (0.39,28.63)	0.58 (0.06,5.36)
Potassium	no	Unadj.	1.77 (0.63,5.00)		1.57 (0.58,4.30)	5.63 (1.90,16.65)**	3.64 (1.22,10.83)*	12.97 (1.61,104.43)*	5.10 (0.63,40.94)
		Adj.	1.05 (0.42,2.61)		1.30 (0.53,3.23)	5.01 (1.59,15.78)*	3.74 (1.19,11.75)*	13.06 (1.32,129.23)*	6.50 (0.69,61.32)
	yes	Unadj.	3.18 (1.23,8.20)*		2.29 (0.89,5.85)	4.68 (1.65,12.27)**	2.70 (0.94,7.75)	20.16 (2.42,168.18)*	7.17 (0.87,59.45)
		Adj.	2.31 (0.82,6.54)		1.60 (0.57,4.48)	3.65 (1.21,10.98)*	2.02 (0.66.6.13)	13.36 (1.35,131.92)*	3.65 (0.37,35.61)

*Abbreviations* Adj.: model adjusted by smoking status, CI: confidence interval, GP: generalized periodontitis, LP: localized periodontitis, OR: odds ratio, T1: first (the lowest) tertile of intake, T2: second tertile of intake, T3: third tertile (the highest) of intake, Unadj.: unadjusted model

* indicates statistical differences between tertiles at p-value < 0.05

** indicates statistical differences between tertiles at p-value < 0.005

Ordered logistic modeling also resulted in similar findings produced by standard logistic regression for the lowest terciles of fiber, potassium, and vitamin B6 intake, compared to the highest terciles, as well as the second tercile of caloric intake. In an unadjusted model, the lowest terciles of fiber and potassium consumption non-normalized and normalized by energy intake were associated with increased odds of having more generalized periodontal disease compared with the highest fiber and potassium consumption tercile, respectively. This association was also statistically significant for non-adjusted intake of vitamin C normalized by energy intake and vitamin B6 consumption non-normalized by energy intake. Likewise, the second tercile of energy intake was associated with increased odds of having more GP. In a model controlling for smoking status, only the lowest terciles of fiber and potassium intake normalized by caloric consumption were associated with increasing extension of periodontitis despite this association also was found for vitamin B6 and potassium non-normalized consumption.

## Discussion

In the present study, periodontal health has been positively related to the dietary intake of fiber, vitamin C, and potassium. In general, inverse associations have been previously reported between intake of dietary fiber [18, 19], potassium [20], and vitamin C [19–22] and any form of periodontal disease. Similarly, the inverse association between fiber intake and vitamin C and the risk of periodontal disease has been confirmed by a, meta-analysis that includes nine publications in community-dwelling older adults [23] but this was also found for fatty acids, vitamin C and E, and calcium intake. In turn, the risk of periodontal disease was higher in individuals with the highest sugar intake in a meta-analysis evaluating the possible role of the role of dietary patterns in periodontal health, including nine studies [24]. Similarly, dietary intake of vitamin E [20], but also carbohydrates [25], phosphorus [21, 25], iron and vitamins B6 and B12 [20] were also inversely associated with any form of periodontal disease by at least one of the cited studies. Interestingly, the association with vitamin B9 intake was the most common relationship reported in such studies [19–21]. Additional studies also reported inverse associations with total fat dietary intakes [26, 27], polyunsaturated fat [28] and calcium [29], but also vitamin A [30], B1 [31] vitamin B2 [30, 31], vitamin B3 [31, 32] and confirmed results for vitamin B9 [33], and iron [20, 32]. However, the association found for the incidence of periodontitis with vitamin B1, and E has been reported to be nonlinear in one of the studies [30]. Therefore, many of the previously reported associations with periodontitis have not been observed in the population of the present study. Regarding vitamin C intake, observational studies are less clear., Some cross-sectional studies found a significant association present only in certain strata [32, 34, 35]. In fact, it has been suggested that age and smoking may be related to the efficacy of vitamin C for the prevention of periodontal disease, but this can depend on the characteristics of the population [5]. However, Jeong et al. [24] reported a higher risk of periodontal disease in the lowest vitamin C intake group after performing a meta-analysis to evaluate the possible role of diet patterns in periodontal health that included nine studies. Interestingly, a more recent study found a nonlinear association between vitamin C intake and periodontitis prevalence. According to this, excessive vitamin C intake would increase the risk of periodontitis [30].

Among the studies mentioned, some of them concomitantly evaluated associations between periodontal health and multiple dietary intakes [20, 21, 26, 27, 30]. As in the present study, none of the mentioned studies found statistically significant associations between periodontal health and dietary intake of proteins, cholesterol, calcium, magnesium, selenium, zinc, and vitamins B1, B12 and K, although this also occurred with fiber and potassium. Other studies evaluating association with the intake of one or two nutrients also reported statistically significant results. This occurred with sugars [36–38] and vitamin C [35] and D [39, 40], but also calcium [29, 41, 42] and magnesium [43]. Moreover, two cross-sectional studies focused on dietary fiber intake [18, 44] in a sample of 5,052 individuals from NHANES 2009–2010 and 2011–2012, supporting the results of the present study. In contrast, a study in non-smokers subjects [41] reported no correlation between calcium intake and the CPI score, although it was inversely associated with the percentage of sites with BOP. Similarly, no association has been previously found between folate intake and CPI, although it suggested that it influences BOP [33].

It is possible that associations between nutritional intake and periodontal disease only appeared when individual with or without nutritional deficiencies and are compared. In these cases, such associations could only be detected in subjects with very low dietary intakes. Some studies evaluated the possible associations of inadequate intake according to the proper nutritional recommendations. In this sense, it has been suggested that the risk of periodontitis was reduced with sufficient intake of vitamin A, B1, B2, and E [30]. Inappropriate vitamin B3 intake also showed higher odds of periodontitis in other study and this association was stronger in women and participants aged 40–59 years. However, this was not found for calcium, vitamin A, B1, B2, and C, and iron intake below the recommendations [31]. Similarly, in the cross-sectional study by Park et al. [32] in young adults, inadequate vitamin B3 intake was significantly associated with periodontitis, which also occurred in the subgroups of women and current non-smokers. Furthermore, calcium nutritional deficiencies have been reported to increase susceptibility to periodontal disease. Calcium intake within age-specific recommendations was associated with a lower likelihood of severe periodontitis in adult participants of the Danish Health Examination Survey (DANHES) 2007–2008 [42].

In the present study, periodontitis was diagnosed according to the stage III and IV definition of the 2017 World Workshop on the Classification of Periodontal and periimplant Diseases and Conditions [11]. According to this diagnose our data show that the values of PD and CAL show the highest values in GP compared to LP and the lowest in NoP. Many cross-sectional studies categorized their samples into patients with moderate to severe periodontitis and those without periodontitis or a less severe form of this disease [18, 30]. Furthermore, the possible association with the severity of the disease that classifies individuals into different degrees of periodontitis has also been evaluated [21]. Sometimes, both strategies have been combined [18]. Among studies conducting alterative comparisons, the associations reached statistical significance when patients with more severe disease were compared with the rest of the population, including health attendants and patients with a mild or slight form of the disease. In particular, this occurred in the study by Nielsen et al. [18] who reported suggesting an association between the risk of severe periodontitis, but also the severity of periodontitis and the fiber intake. As here, the high prevalence of periodontitis and the extension of periodontitis in the analyzed population could condition the absence of significant associations with mild to severe periodontitis. In fact, mild or no periodontitis were grouped into the referent category to mitigate the risk of bias due to a potentially excessive prevalence of mild periodontitis in the population [45].

The associations between clinical parameters and dietary intake also evidenced the relationship between nutritional intake and the severity of periodontal disease. However, more ancient studies using additional clinical variables usually did not find many differences between nutritional intake categories or correlations with nutritional intake. No correlation was found between RPI (which scored the presence and severity of gingival bleeding and pocket depth) and the dietary intakes of phosphorus and potassium, but also iron, calcium vitamin A, B1, B2, B3, in patients from a dental clinic [46]. Another study in non-smokers subjects [41] reported no correlation between calcium intake and CPI score, although it was inversely associated with percentage of sites with BOP. Similarly, no association has been previously found between folate intake and CPI despite of it being suggested that it has an effect on BOP [33]. Here, differences in the proportion of site with a PD of 4 mm or more supported the role of fiber and potassium, but without other parameters. Therefore, choosing the appropriate oral health-related parameter to compare nutritional intake groups is a critical point.

However, if total energy intake is associated with the outcome, the adjustment for it is important in epidemiological analyses because it can operate as a confounding variable since intakes of most nutrients are associated with energy intake, often strongly. Even, it has been suggested that, if not associated with the outcome, differences in total energy intake can result in extraneous variation in nutrient intake due to individual differences in physical activity, body size, and metabolic efficiency. In this situation, failure to adjust for total energy intake can lead to misclassification of biologically important variation in nutrient intake and result in attenuation of associations [47, 48]. In the present study, the residual model that indirectly adjusts for total energy by using a residual was used to adjust for energy intake [47]. This estimates the average relative causal effect of total energy intake but provides biased estimates even in the absence of confounding. In the study that reported associations for potassium, but also iron and vitamins B6 and B12, single nutrient intakes were expressed as mean intakes per 1000 kcal of total energy [20]. A similar adjustment was made by Hamasaki et al. [26]. However, in most of the studies mentioned, no energy adjustment method was described [18, 21, 30, 32]. Moreover, the difficulties in interpreting the “nutrient density model” have been previously pointed out [47]. Differences in the models used or the absence of an adjustment for energy intake may also explain some of the apparent heterogeneity between existing nutritional studies. In fact, the intake of nutrients previously associated with higher odds of having any periodontal disease, at least in severe form, was also associated with severe periodontitis or periodontitis severity here after adjustment by smoking, such as vitamin B1, B2, B6 and iron.

However, the current study presents some limitations. First, the cross-sectional design does not allow for a longitudinal evaluation of the cause-and-effect relationship between exposure and outcome and therefore can only be used to build a hypothesis. Indeed, this design also does not allow us to properly investigate reverse causality (i.e., effect of periodontitis on nutritional capacity and dietary changes). Furthermore, no molecular parameters supporting the biological plausibility of such an association were registered. Second, FFQ estimated the nutrient and energy dietary intake to be not as accurate as other quantitative dietary assessment methods, and the usual portion size questions are prone to measurement error since they are based on generic memory [49]. This error can be at least partially mitigated by using a less biased dietary assessment method as a reference instrument. Importantly, FFQs would be less affected by reactivity, and if they are shorter FFQs, as in this case, they will have higher response rates and lower respondent burdens [50]. Unfortunately, such information only represents the current diet, which along with possible memory errors and bias may not represent the real nutritional status of the sample. Furthermore, FFQ collected dietary data using a context-specific food list to estimate the usual diet, and additional measurement errors can be introduced when food lists are not specific to the studied population and when they use inconsistent or imprecise portion sizes [51] or when food lists are not granular enough to make an accurate match to a food composition table to derive the nutrient content of the diet. However, FFQs were adapted and validated instruments for the context of this study were used. Furthermore, since the study population was selected among patients attending the Dental School of the University of Sevilla, the risk of selection bias could not be ruled out, which can reduce the generalizability of the study.

## Conclusions

This study found a significant association between dietary intake of energy, fiber, potassium, and vitamin C intake and generalized periodontitis, as well as the extension of periodontitis. To our knowledge, this was the first study to report an inverse association between potassium intake and periodontitis. Adjusting nutrient intake could help prevent and treat periodontal disease in dental patients. Maintaining an adequate intake of fiber, potassium, and vitamin C could be important to controlling periodontitis. However, due to the cross-sectional design of the present study, at least new additional cohort studies should be conducted on groups of patients with similar characteristics before establishing any diet guidelines for populations.

## Data Availability

The datasets used and/or analysed during the current study are available from the corresponding author on reasonable request.In Methods is described that the study has been approved by the Local Research Committee Hospital Virgen Macarena Sevilla Spain with the identification number: 1588-N-20, 12-21-2016.

## Abbreviations

BMI: body mass index
PD: probing pocket depth
CAL: clinical attachment level
NoP: nonperiodontitis patients
LP: localized periodontitis
GP: generalized periodontitis
FFQ: semiquantitative food frequency questionnaire
